# *Porphyromonas gingivalis* drives trimethylamine-N-oxide accumulation via modulation of gut microbial trimethylamine lyase in mice

**DOI:** 10.3389/fmicb.2026.1786725

**Published:** 2026-04-07

**Authors:** Weige Xie, Dong Han, Jinlu Tan, Dan Zhao, Jingwen Dong, Juan Wu, Xuebin Yang, Sijing Xie

**Affiliations:** 1 Nanjing Stomatological Hospital, Affiliated Hospital of Medical School, Institute of Stomatology, Nanjing University, Nanjing, China; 2 Division of Oral Biology, School of Dentistry, University of Leeds, Leeds, United Kingdom

**Keywords:** choline, gut microbiota, *Porphyromonas gingivalis*, trimethylamine-lyase, trimethylamine-N-oxide

## Abstract

**Introduction:**

Trimethylamine N-oxide (TMAO), a gut microbiota-derived metabolite, is linked to cardiovascular, neurodegenerative, and metabolic diseases. Emerging evidence indicates a bidirectional interaction between the periodontal pathogen *Porphyromonas gingivalis* (*Pg*) and gut microbiota, potentially influencing host TMAO metabolism. However, whether *Pg* modulates the choline-trimethylamine (TMA) axis remains unclear.

**Methods:**

Wild-type male C57BL/6J mice received oral *Pg* under chow or a high-choline diet. Plasma and cecal concentrations of TMA and TMAO were quantified, intestinal barrier function was evaluated via histological analysis, and the determination of ZO-1 and occludin expression was performed. Cecal microbiota composition was profiled by 16S rRNA gene sequencing, and microbial choline-TMA lyase markers (*cutC*/*cutD*) were measured.

**Results:**

*Pg* elevated plasma TMAO under chow, accompanied by reduced *α*-diversity, altered *β*-diversity, and decreased expression of intestinal barrier proteins. Under high-choline conditions, the diet itself increased plasma and intestinal levels of TMAO and TMA. *Pg* co-exposure further amplified these effects, raising plasma TMAO, cecal TMA, and *cutC*/*cutD* levels. Microbiome analysis revealed elevated abundances of *Lachnoclostridium*, *Odoribacter*, and *Colidextribacter*, and reduced levels of taxa (*Prevotellaceae NK3B31*, *Anaerostipes*, and *Ruminococcus*) negatively correlated with TMAO-related parameters. Moreover, *cutC*/*cutD* levels were positively correlated with *Colidextribacter* and *Lachnoclostridium*, but negatively correlated with *Anaerostipes* and *Prevotellaceae NK3B31*, consistent with the modulation of TMA/TMAO metabolism by these taxa.

**Conclusion:**

This study demonstrates that oral administration of *Pg* facilitates systemic TMAO elevation by reshaping gut microbial communities and enhancing choline-TMA lyase function, and compromising intestinal barrier integrity. These findings establish an oral-gut metabolic axis connecting periodontitis to host TMAO metabolism, and highlight promising periodontal and microbiota-targeted strategies for alleviating TMAO-associated systemic disorders.

## 1 Introduction

Trimethylamine N-oxide (TMAO), an organic amine oxide (CH₃)₃NO, is a gut microbiota-derived metabolite that has emerged as a key factor associated with multiple systemic diseases (Janeiro et al., 2018), including cardiovascular, neurodegenerative, and metabolic disorders (Caradonna et al., 2025; Thomas and Fernandez, 2021). In the human body, TMAO is formed through a two-step metabolic process: intestinal microbes convert dietary precursors such as choline and L-carnitine into trimethylamine (TMA), which is then absorbed into the portal circulation and oxidized to form TMAO in the liver, primarily by flavin-containing monooxygenases 3 (FMO_3_) (Loo et al., 2022). It was reported that elevated circulating TMAO levels have been linked to a variety of systemic diseases, including atherosclerosis (AS), chronic kidney disease, colorectal cancer, and Alzheimer’s disease, positioning TMAO metabolism at the interface between the gut microbiota, diet, and systemic health (Albulushi and Taha, 2025; Jiang et al., 2025; Saha et al., 2025; Su et al., 2025).

Periodontitis is a common chronic inflammatory oral disease driven by microbial dysbiosis within the subgingival niche and characterized by gingival bleeding, periodontal pocket formation, and eventual tooth loss. Among key pathogens, *Porphyromonas gingivalis*(*Pg*) is considered a core member of the oral “red complex” (with *Tannerella forsythia* and *Treponema denticola*) (Demkovych et al., 2023), exhibiting potential virulence through lipopolysaccharide (LPS), gingipains, and outer membrane vesicles, which enable host immune evasion and persistent chronic inflammation (Matrishin et al., 2024; Nemoto and Ohara, 2021). Notably, epidemiological and experimental evidence suggest that *Pg* contributes to TMAO-related systemic diseases, including cardiovascular disease (CVD) progression and non-alcoholic fatty liver disease (NAFLD), via both direct hepatic tissue colonization and indirect induction of gut dysbiosis (Bahitham et al., 2025; Budoff et al., 2025; Zhong et al., 2025).

Recent studies, including our own work, indicated a link between periodontitis and elevated plasmaTMAO levels. We previously demonstrated that oral administration of *Pg* accelerates AS in an apolipoprotein E-deficient (*Apoe^−/−^*) mouse with increased plasma TMAO levels (Xiao et al., 2021). Moreover, we compared the modulatory effects of three common oral bacterial strains, including *Pg*, *Fusobacterium nucleatum* (*F. nucleatum*), and *Streptococcus mutans* (*S. mutans*), and found that only *Pg* significantly increased plasma TMAO in C57BL/6J mice, suggesting a unique role of *Pg* in regulating TMAO metabolism (Wang et al., 2024). Also, nonsurgical periodontal therapy (NSPT) significantly reduced the periodontitis-elicited elevation of plasma TMAO levels in *Apoe^−/−^* mice (Huang et al., 2025). These studies have progressively clarified that *Pg* is an important driver of TMAO metabolic disorders in AS, and its mechanism of action mainly targets gut-liver crosstalk.

These findings were consistent with Zhou et al. (2022), who reported that patients with stage III-IV periodontitis had significantly higher TMAO levels accompanied by vascular endothelial dysfunction; however, nonsurgical periodontal treatment could normalize plasma TMAO levels, improve vascular endothelial function, and reduce systemic inflammation. Gan et al. (2023) also confirmed that chronic apical periodontitis induced by pulp implantation of *Pg* increased serum TMAO in *Apoe^−/−^* mice, and TMAO levels were positively correlated with the severity of atherosclerotic lesions. However, *Apoe^−/−^* murine models have inherent intrinsic lipid metabolic and gut microbiota disturbances, limiting mechanistic clarity. Therefore, a critical question remains: does *Pg* directly influence gut microbial TMA production independently in metabolically normal mice, and through which pathways?

To address these research gaps, this study investigated whether oral exposure to *Pg* alters plasma TMAO levels and gut microbiota composition in metabolically normal wild-type C57BL/6J mice, and characterized associated changes in gut microbiota composition and intestinal mucosal barrier integrity. In addition, it was further clarified whether these effects are amplified under a high-choline diet designed to enhance the Choline-TMA-TMAO metabolic axis. Then, the effects of *Pg* on gut microbial choline-TMA lyase (*cutC*/*cutD*) activity were also examined to reveal a mechanistic link between periodontal *Pg* infection and systemic TMAO-related metabolic dysregulation, thereby providing theoretical insights for preventing and managing systemic diseases mediated by periodontal pathogens.

## 2 Materials and methods

### 2.1 Experimental animals

All experimental procedures were approved by the Institutional Animal Care and Use Committee (IACUC) of Nanjing University (IACUC No. IACUC-D2102033) and conducted in accordance with ARRIVE guidelines. Six-week-old male C57BL/6J mice were obtained from the Model Animal Research Center of Nanjing University and housed under specific pathogen-free (SPF) facilities with a 12 h light/dark cycle, and free access to water and food for 2 weeks’ acclimation before any experimental procedure.

Experiment 1: Twelve 6-week-old mice were fed a basal diet for 2 weeks for acclimatization, then randomly to Control (Con: standard chow diet, 10% fat, 0.1% choline) with sterile carboxymethyl cellulose (CMC) slurry around the right maxillary second molar (M2) or *Pg* group (*P*g: same diet) with periodontitis induced by ligating the right maxillary M2 and applying CMC slurry containing *Pg*. At week 8, the mice were intraperitoneally administered pentobarbital sodium at a dose of 50 mg/kg for preliminary anesthesia, and then subjected to cervical dislocation to confirm euthanasia. The plasma, cecal contents, small intestine, large intestine, and liver tissues of the mice were harvested for subsequent analysis.

Experiment 2: Fifteen 6-week-old mice were fed a basal diet for 2 weeks for acclimatization, then randomly divided into three groups: Con (standard chow diet and sterile CMC slurry), Choline (standard chow diet supplemented with 1% choline and sterile CMC slurry), or Choline + *P*g (standard chow diet supplemented with 1% choline and CMC slurry). Applications were performed five times per week for 6 weeks. All mice were euthanized humanely at week 8 for tissue sample collection.

### 2.2 Application of *Pg* slurry

*Pg* strain 33,277 was cultured anaerobically (37 °C for 48 h) inside an anaerobic jar (Oxoid, England) in Brain Heart Infusion Broth (Beyotime Biotechnology, China) under strict anaerobic conditions (AnaeroPack system, Mitsubishi Gas Chemical, Japan). Bacterial density was quantified by spectrophotometric analysis (SpectraMax M3, Molecular Devices, USA). At an optical density (OD) of 600 nm (OD₆₀₀), corresponding to 1 × 10^9^ colony-forming units (CFU) per millilitre (CFU/mL) (Sakanaka et al., 2016), *Pg* were harvested and resuspended in 100 mL inphosphate-buffered saline (PBS) supplemented with 2% CMC (Sigma-Aldrich, USA) (Kato et al., 2018). Mice in the *P*g group received 100 μL CMC slurry containing 5 × 10^6^ CFU *Pg* around the cervical margin of the right maxillary second molar (M2), five times weekly (Gan et al., 2023). Mice in the Control groups received an equal volume of sterile CMC slurry without *Pg*. In Experiment 2, the Choline + *P*g group received identical *Pg* inoculation procedures, while the Con and Choline groups were treated with *P*g bacteria-free CMC slurry.

### 2.3 UHPLC–MS/MS of plasma and cecal TMAO-related parameters

TMAO, TMA, choline, creatinine, betaine, and L-Carnitine were quantified using stable isotope-dilution ultra-high performance liquid chromatography–tandem mass spectrometry (UHPLC–MS/MS) (Jia et al., 2020). Separation used a Waters ACQUITY UPLC HSS T3 column (2.1 × 100 mm, 1.8 μm) with mobile phases A (0.1% formic acid in water) and B (0.1% formic acid in acetonitrile). The MS quadrupole and ion source temperatures were 100 °C and 650 °C. Multiple reaction monitoring (MRM) transitions are listed in Table 1 (Goh et al., 2021; Koeth et al., 2019).

**Table 1:** Multiple reaction monitoring (MRM) transitions used for UHPLC–MS/MS quantification.

Analyte	Precursor ion (m/z)	Product ion (m/z)
TMAO	76.1	58.1
TMA	60.0	40.5
Betaine	118.1	58.1
Choline	104.1	45.0
Creatinine	114.1	44.0
L-Carnitine	162.1	85.0

### 2.4 Gut microbial metagenomic DNA extraction and microbial diversity analysis

Mice cecal content samples were collected and immediately frozen at −80 °C. Total DNA was extracted using the TIANamp Stool DNA Kit (Tiangen, China) according to the manufacturer’s instructions (Jameson et al., 2018). A bacterial 16S ribosomal RNA (rRNA) gene sequencing assay was performed to investigate the microbiota community composition in the cecum. Genomic DNA was used as a template to amplify the 16S rRNA V3–V4 region, which serves as a molecular marker for bacterial diversity analysis.

After removal of barcodes and primers, the obtained sequences were clustered into the operational taxonomic units (OTUs) at a 97% similarity threshold and classified against the SILVA128/16S bacte*ria data*base for taxonomic assignment (Jameson et al., 2018). Bioinformatics analysis of microbial diversity was performed using the Illumina HiSeq platform at the Novogene Bioinformatics Institute (Beijing, China) (Dong et al., 2022).

### 2.5 Real-time polymerase chain reaction analysis

Total RNA from liver and small intestine tissues was extracted using RNA simple Total RNA Kit (Tiangen, China) and reverse-transcribed. qPCR was run using the PCR Thermal Cyclers (Applied Biosystems, Thermo Fisher, America) (Ramireddy et al., 2021). Primer sequences are listed in Table 2.

**Table 2:** Primer sequences used for RT-qPCR.

Gene	Primer sequences (5′ → 3′)
IL-6	F: TCTATACCACTTCACAAGTCGGA R: GAATTGCCATTGCACAACTCTTT
IL-1β	F: GAAATGCCACCTTTTGACAGTG R: TGGATGCTCTCATCAGGACAG
TNF-α	F: CTGAACTTCGGGGTGATCGG R: GGCTTGTCACTCGAATTTTGAGA
TNF-β	F: TTCTCCGACATGGCTTCTCTT R: TGGAAGGGGTATTGGGAGGAA
IL-10	F: GCTGGACAACATACTGCTAACC R: ATTTCCGATAAGGCTTGGCAA
ZO-1	F: GCCGCTAAGAGCACAGCAA R: GCCCTCCTTTTAACACATCAGA
Occludin	F: TGAAAGTCCACCTCCTTACAGA R: CCGGATAAAAAGAGTACGCTGG
FMO3	F: GGCCTGTGGAAATTCTCAGAC R: AAGTCATCGGGATAGGGGAAG
GAPDH	F: AGGTCGGTGTGAACGGATTTG R: GGGGTCGTTGATGGCAACA

### 2.6 Histological analysis of intestinal and liver tissue

Intestinal and hepatic tissues were fixed in 4% paraformaldehyde for 24 h, embedded in paraffin, sectioned (5 μm), and stained with hematoxylin and eosin (HE). Images were captured on a Manoramic Mid scanner (3DHISTECH, Hungary) (Camilleri et al., 2012).

ZO-1 immunofluorescence was performed using rabbit anti-ZO-1 antibody (1:100, 21773-1-AP, Proteintech, USA) and Alexa Fluor 488 goat anti-rabbit IgG secondary antibody (1:200, GB25303, Servicebio, China). Primary rabbit anti-occludin antibody (1:300, 27260-1-AP, Proteintech, USA) was added to the samples and incubated overnight at 4 °C. After three washes with PBS (5 min each), fluorescent secondary antibodies (1:200, GB21303, Servicebio, China) were applied. Fluorescent images were captured from three microscopic fields per mouse (*n* = 3) using a Nikon Ti confocal laser scanning microscopy (Nikon, Japan) and quantified using ImageJ software (National Institutes of Health, USA) (Flak et al., 2022).

### 2.7 *CutC*/*cutD* protein detection

The protein levels of cecal glycyl radical enzyme (*cutC*) and glycyl radical-activating protein (*cutD*) were measured by using commercial ELISA kits (JM-0224W1, JM-0238W1, Jingmei Biotechnology Co., Ltd., Nanjing, China).

### 2.8 Statistical analysis

All data are presented as mean ± standard deviation (SD). Group differences were assessed using *t-*tests or one-way ANOVA with *post hoc* tests as appropriate. Correlation between bacterial taxa and TMAO-related parameters was evaluated using Spearman’s correlation coefficient test. Statistical analyses were performed using SPSS Statistics v26.0 (IBM Corp., USA), with *p* < 0.05 considered statistically significant.

## 3 Results

### 3.1 Oral application of *Pg* increases plasma TMAO metabolites in C57BL/6J mice

**Figure 1:**
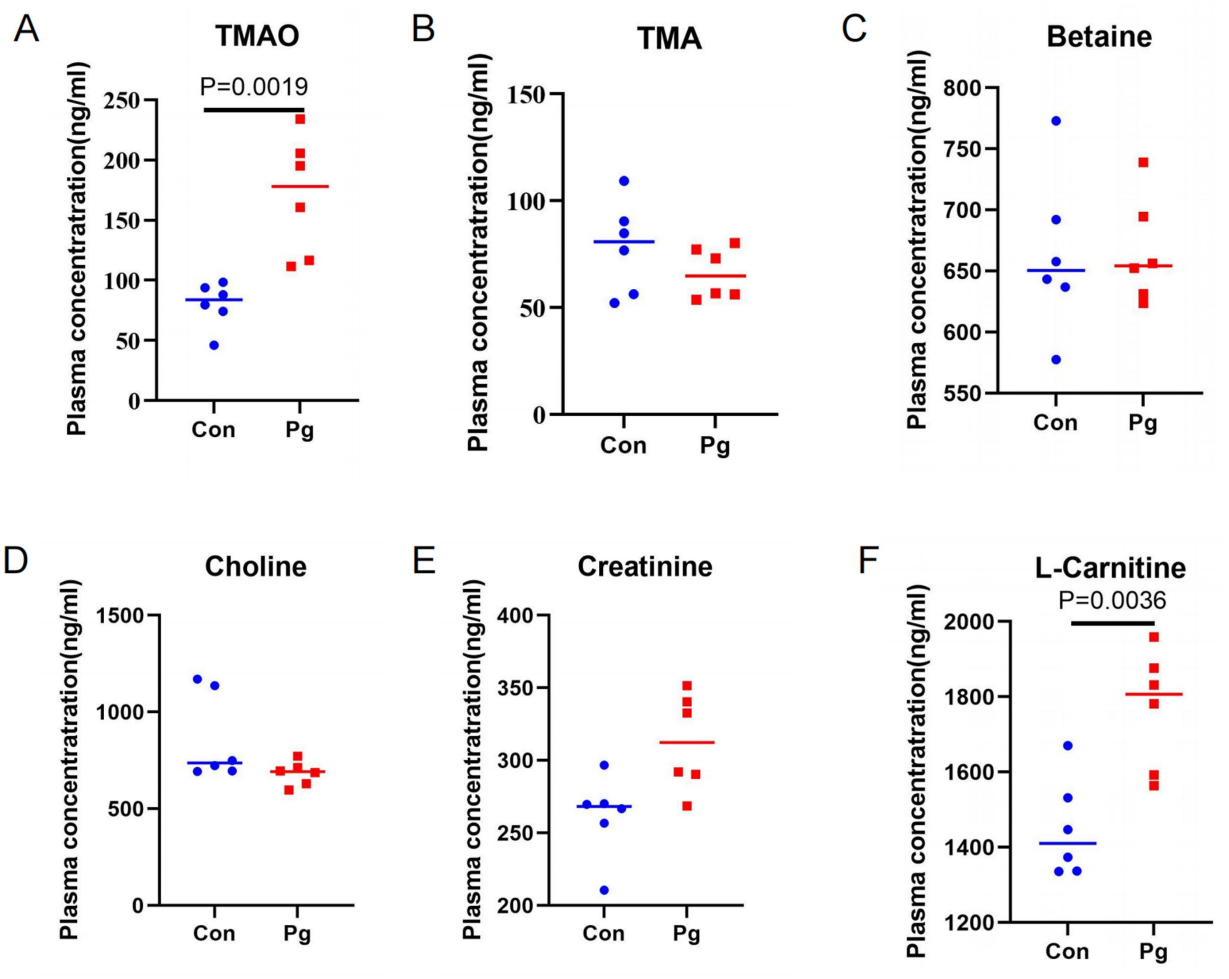
Plasma concentrations of TMAO and related metabolites of C57BL/6J mice: **(A)** Plasma TMAO concentration; **(B)** Plasma TMA concentration; **(C)** Plasma betaine concentration; **(D)** Plasma choline concentration; **(E)** Plasma creatinine concentration; **(F)** Plasma L-carnitine concentration. Data are presented as mean ± SD (*n* = 6 mice per group).

LC–MS/MS quantification results (Figures 1A–F) showed that *Pg* significantly increased plasma TMAO concentrations compared with the Con group, whereas plasma TMA levels showed no significant difference. Among TMA precursors, plasma creatinine and L-carnitine concentrations were significantly increased in the *P*g group, while betaine and choline remained unchanged.

### 3.2 Oral *Pg* administration impairs the intestinal mucosal barrier in C57BL/6J mice

**Figure 2:**
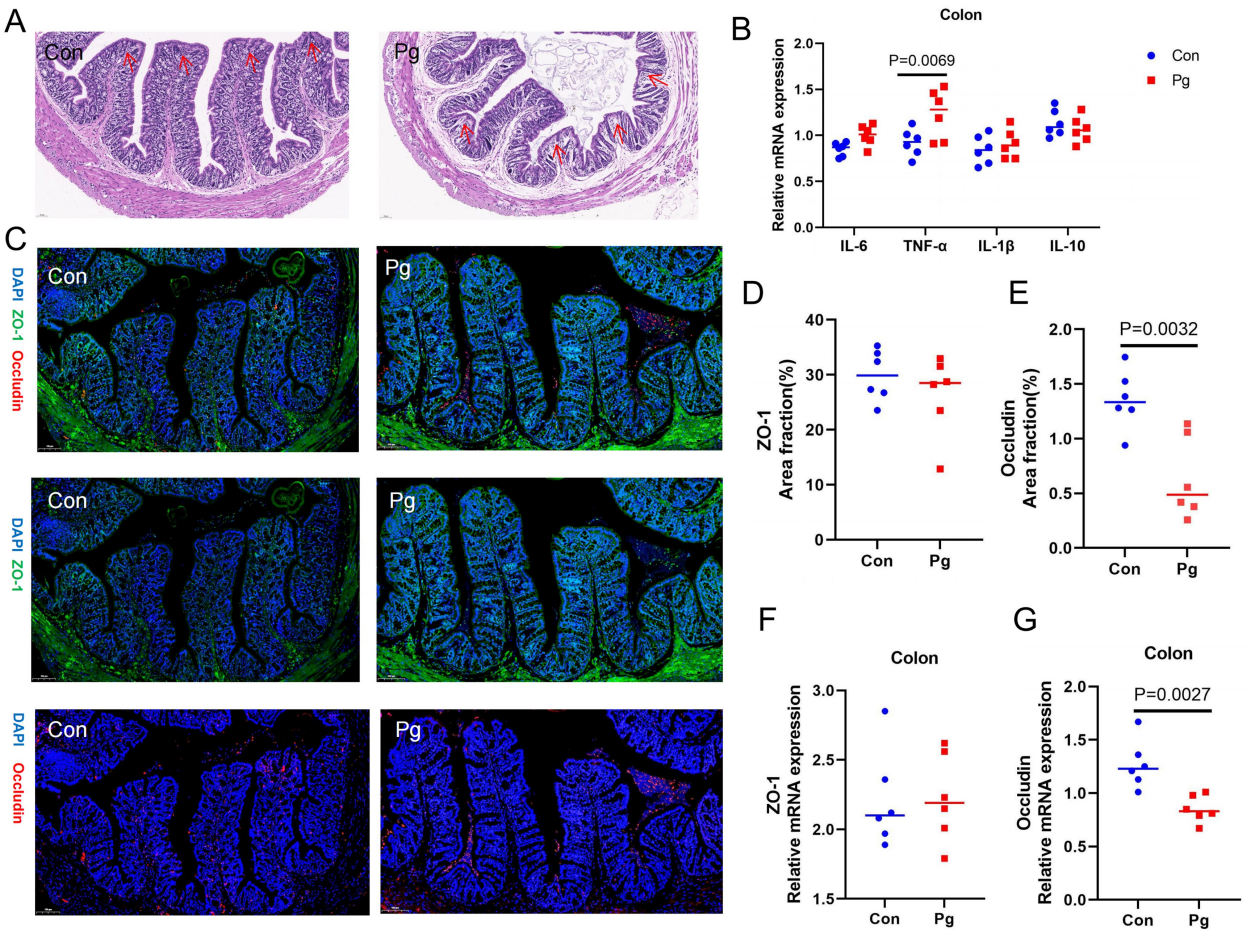
Effects of *Pg* on intestinal mucosal barrier. **(A)** Hematoxylin and eosin staining of the colon. Scale bar = 50 μm. **(B)** The mRNA expression of IL-6, TNF-α, IL-1β, and IL-10 in colon tissue. **(C–E)** Representative images of ZO-1 and occludin immunostaining **(C)** and quantification of area fractions in the colon **(D,E)**. Scale bar = 100 μm. **(F,G)** Relative mRNA expressions of ZO-1 and occludin. Data are presented as mean ± SD (*n* = 6 mice per group).

HE staining revealed greater colonic inflammation (e.g., increased inflammatory cell infiltration and crypt damage) in the *P*g group compared with the Con group (Figure 2A). The TNF-α mRNA expression in the colon tissue was markedly upregulated in *P*g mice compared to that in the Con group (Figure 2B). Immunofluorescence staining demonstrated a significant reduction in occludin expression in the *P*g group (Figures 2C,E), which was further confirmed by mRNA (Figure 2G), consistent with barrier impairment. However, there were not statistical significant on ZO-1 expression between the two groups (Figures 2D,F).

### 3.3 Oral *Pg* administration alters gut microbiota composition in C57BL/6J mice

**Figure 3:**
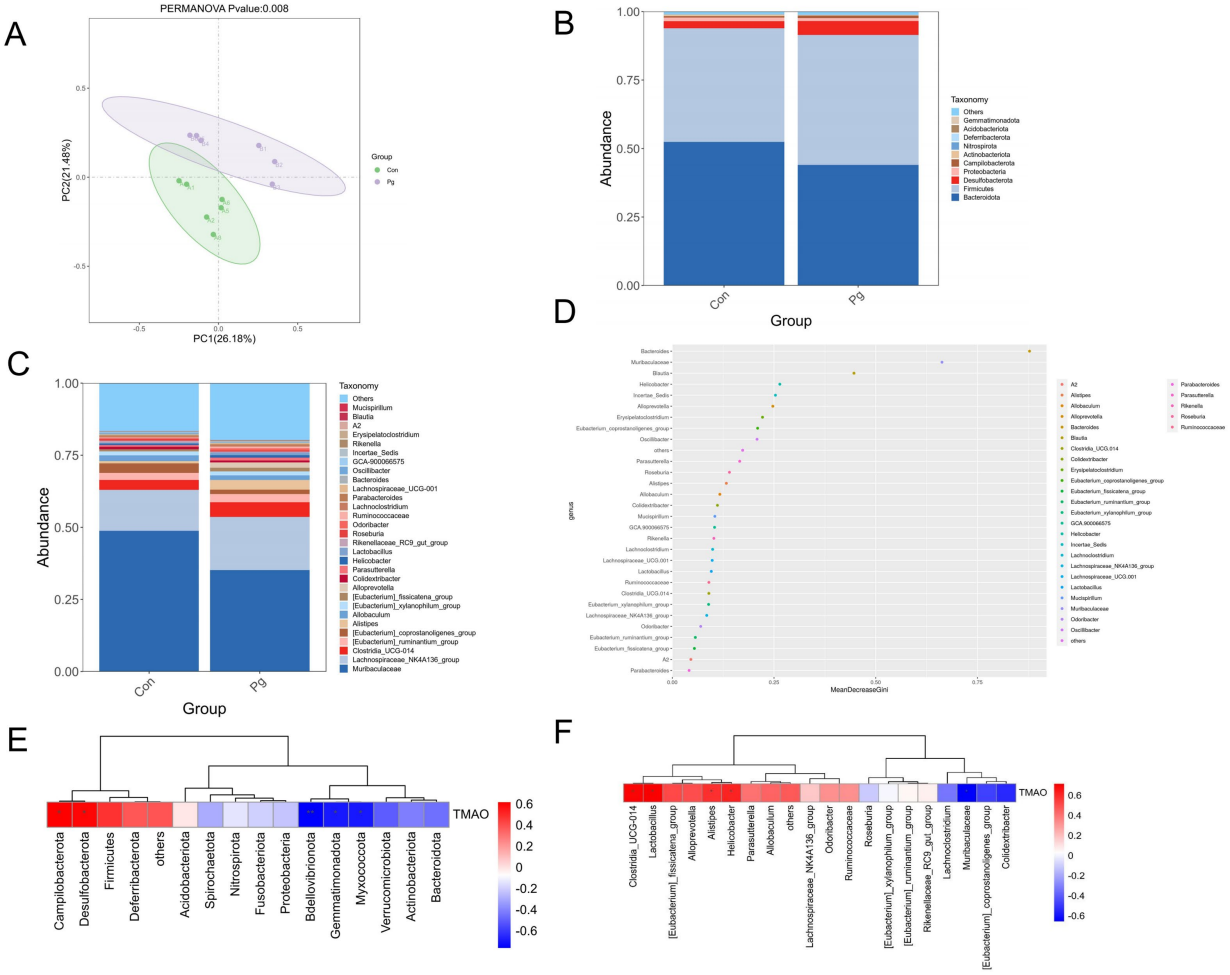
Effects of oral *P*g on gut microbiota composition. **(A)** Principle coordinate analysis (PCoA) based on Bray Curtis distance; **(B)** phylum-level taxonoic differences of intestinal flora; **(C)** genus-level differences of intestinal flora; **(D)** random forest analysis identifying key discriminatory genera; **(E)** correlation between top 20 phyla and TMAO parameters; and **(F)** correlation between top 20 genus and TMAO parameters based on the Spearman correlation coefficient test (*n* = 6 mice per group). ^*^p < 0.05, ^**^p < 0.01.

16S rRNA gene sequencing and α-diversity analysis showed that *Pg* treatment significantly reduced gut microbial complexity and diversity compared with the Con group, as reflected by lower OTUs, Chao1, Shannon index, and phylogenetic diversity (Supplementary Figure 1A). Principal coordinate analysis (PCoA) based on Bray-Curtis distance revealed distinct separation between the two groups (Figure 3A). Venn diagram analysis identified 225 shared bacterial taxa between groups, with 196 unique taxa in the Con group and 141 unique taxa in the *P*g group (Supplementary Figure 1B).

At the phylum level, *Pg* treatment increased the relative abundance of *Firmicutes* and *Desulfobacterota*, while decreasing *Bacteroidota* (Figure 3B). Compared with the control group, the ratio of F/B in the Pg group increased, but this difference was not statistically significant (Supplementary Figure 1C). At the genus level, *Pg* decreased the relative abundance of *Muribaculaceae*, *Blautia*, *Bacteroides*, and *Oscillibacter*, but increased that of *Helicobacter*, *Clostridia_UCG-014*, *Alistipes*, and *Lactobacillus* (Figure 3C). Random forest analysis further identified Bacteroides, *Muribaculaceae*, *Blautia*, and *Helicobacter* as the key discriminatory genera (Figure 3D).

Spearman correlation analysis based on the top 20 bacterial taxa at the phylum and genus levels was performed. At the phylum level, plasma TMAO levels were positively correlated with *Campylobacterota* and *Desulfobacterota*, but negatively correlated with *Bdellovibrionota*, *Myxococcota*, and *Gemmatimonadota* (*p* < 0.05). At the genus level, plasma TMAO levels were positively correlated with *Clostridia_UCG-014*, *Lactobacillus*, *Alistipes*, and *Helicobacter*, but negatively correlated with *Muribaculaceae* (*p* < 0.05) (Figures 3E,F).

### 3.4 Oral *Pg* administration elevates plasma TMAO and TMA levels, cecal TMA in mice fed a high choline diet

**Figure 4:**
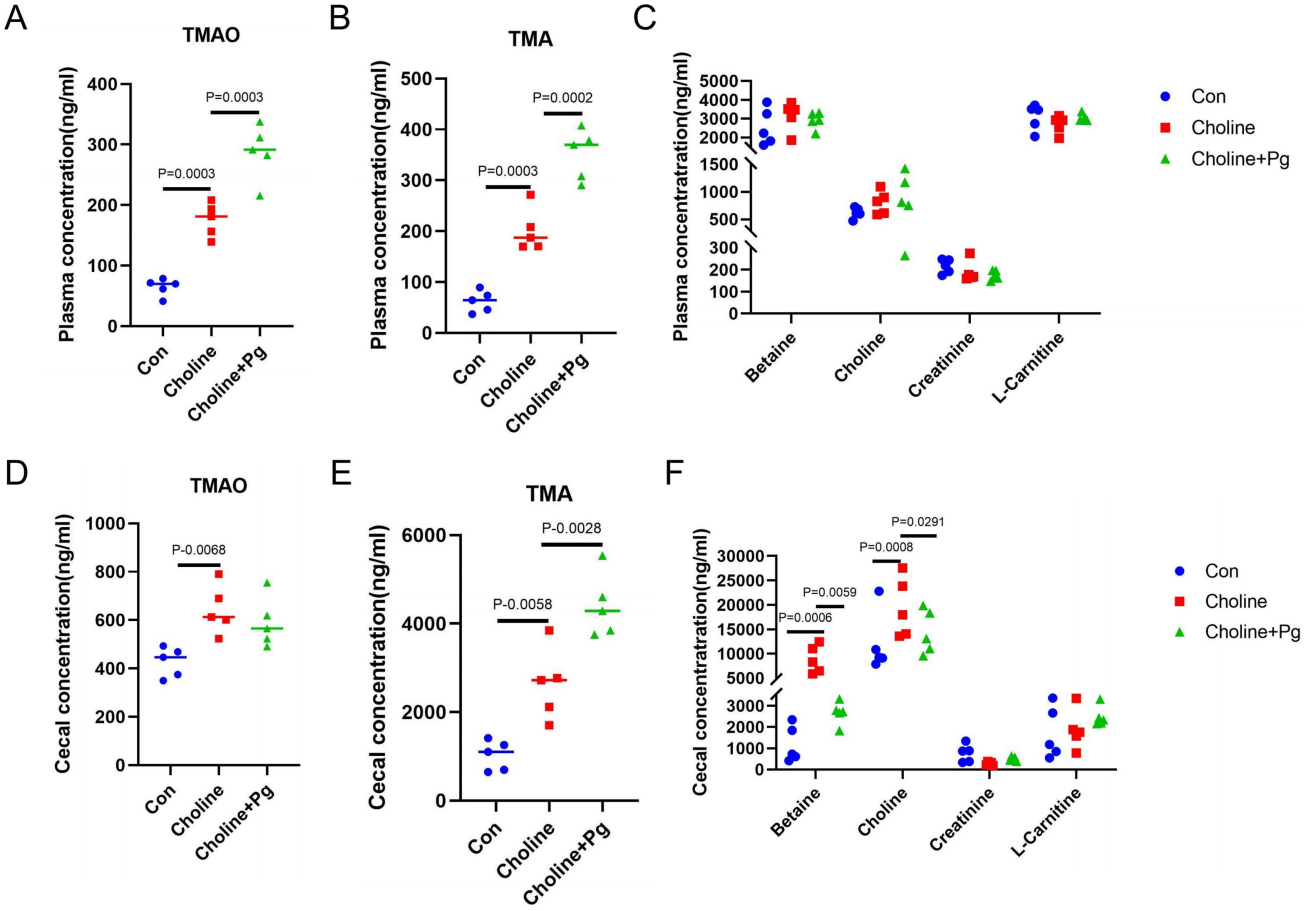
Oral administration of *Pg* elevates plasma and cecal TMAO and TMA levels in mice. **(A)** Plasma TMAO concentrations. **(B)** Plasma TMA concentrations. **(C)** Plasma concentrations of betaine, choline, creatinine, and l-carnitine. **(D)** Cecal TMAO concentrations. **(E)** Cecal TMA concentrations. **(F)** Cecal concentrations of betaine, choline, creatinine, and l-carnitine. Data are presented as mean ± SD (*n* = 5 mice per group). Statistical significance was determined by one-way ANOVA followed by Tukey’s post-hoc test.

Plasma TMAO and TMA concentrations were significantly higher in the Choline group than those in the Con group, and further increased in the Choline + *P*g group (Figures 4A,B). However, other plasma precursors showed no significant differences among the three groups (Figure 4C). In the cecum, TMAO and TMA concentrations were significantly higher in the Choline group compared with the Con, with TMA increased further in Choline + *P*g mice, while TMAO levels remained unchanged (Figures 4D,E). Moreover, both cecal betaine and choline concentrations were significantly higher in the Choline group than in the Con, but decreased upon oral application of *Pg* (Figure 4F). These findings suggest that *Pg* promotes intestinal TMA biosynthesis under high-choline conditions, potentially by enhancing microbial TMA lyase activity.

### 3.5 Effects of oral application of *Pg* on the composition of intestinal microbiota in mice fed a high choline diet

**Figure 5:**
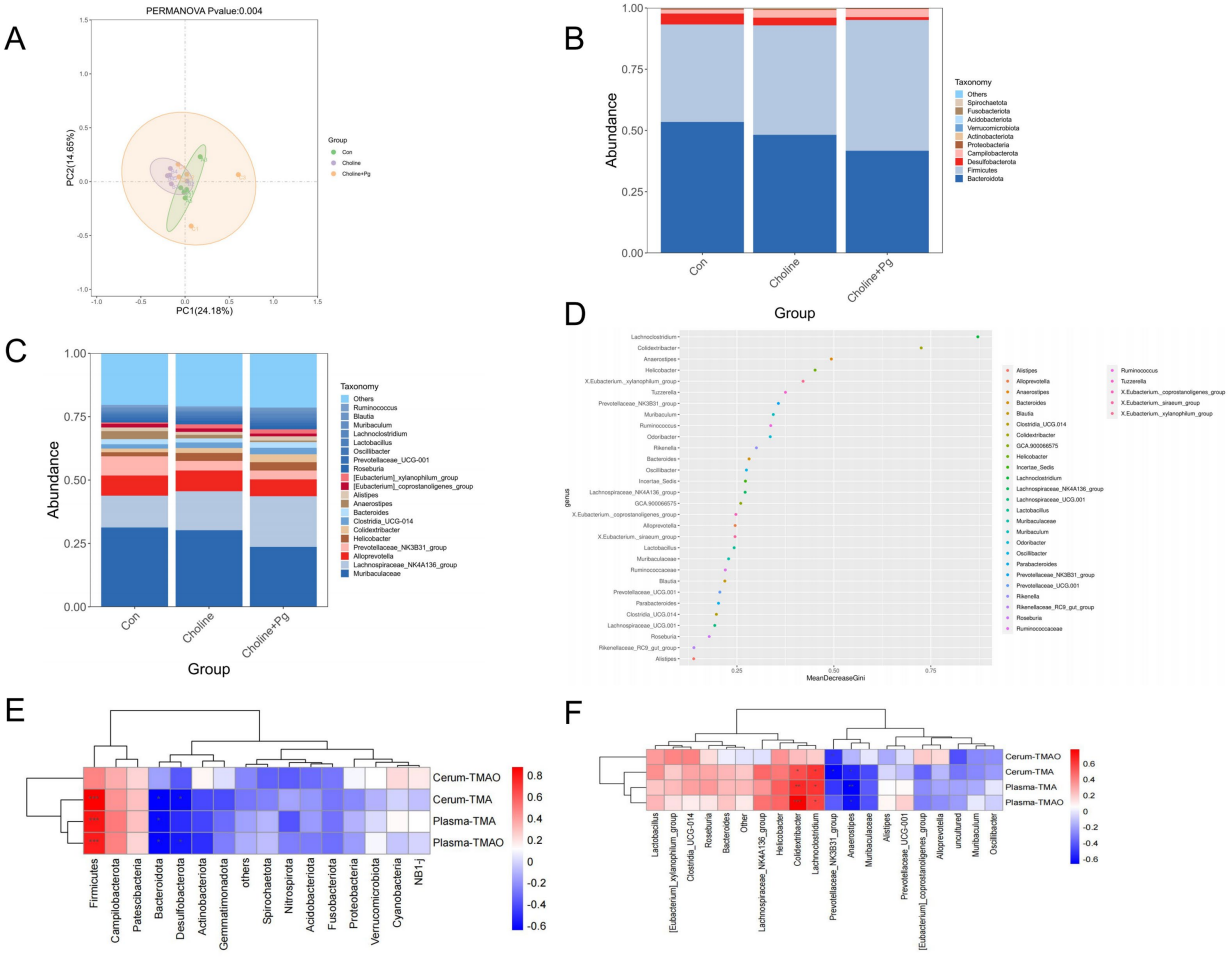
Oral application of *Pg* modulates gut microbiota composition in choline-supplemented mice. **(A)** Principle coordinate analysis (PCoA) of gut microbiota based on Bray-Curtis distance; **(B)** Phylum-level taxonomic differences in intestinal flora; **(C)** Genus-level differences in intestinal flora; **(D)** Random forest analysis of discriminatory genera; **(E)** Spearman correlation coefficient test between top 20 phyla and TMAO-related parameters; **(F)** Spearman correlation coefficient test between top 20 genera and TMAO-related parameters. Data are presented as mean ± SD (*n* = 5 mice per group). *p < 0.05, **p < 0.01, ***p < 0.001.

Alpha-diversity analysis showed no significant differences among the three groups (Supplementary Figure 2A). However, PCoA analysis indicated significant differences in overall microbial community structure among the three groups (Figure 5A). Venn diagram analysis identified 428 common bacterial taxa across all groups. The number of unique taxa was 319 in the Choline + *P*g group, 262 in the Choline group, and 68 in the Con group (Supplementary Figure 2B).

At the phylum level, the Choline + *P*g group exhibited decreased relative abundances of *Bacteroidota* and *Desulfobacterota*, but increased *Firmicutes* compared to the Choline group (Figure 5B). At the genus level, the Choline alone group showed elevated levels of *Odoribacter* and *Colidextribacter*, but reduced levels of *Lachnoclostridium*, *Prevotellaceae-NK3B81-group*, *Anaerostipes*, and *Ruminococcus*. Notably, the combination of choline and *P*g further enhanced the abundances of *Lachnoclostridium*, *Odoribacter*, and *Colidextribacter*, while further reducing *Prevotellaceae-NK3B81-group*, *Anaerostipes*, and *Ruminococcus* (Figure 5C). Random forest analysis identified *Lachnoclostridium*, *Colidextribacter*, *Anaerostipes*, and *Helicobacter* as the key differential genera among the three groups (Figure 5D).

Spearman correlation analysis showed that, at the phylum level, *Firmicutes* was positively correlated with plasma TMAO, plasma TMA, and cecal TMA, whereas *Bacteroidota* was negatively correlated with plasma TMAO (Figure 5E). At the genus level, *Lachnoclostridium*, *Odoribacter*, and *Colidextribacter* were positively correlated with most TMAO-related indicators, whereas *Prevotellaceae-NK3B81-group*, *Anaerostipes*, and *Ruminococcus* showed strong negative correlations (Figure 5F).

### 3.6 Oral *Pg* administration increases choline-TMA lyase (*cutC*/*cutD*) expression in mice fed with a high-choline diet

**Figure 6:**
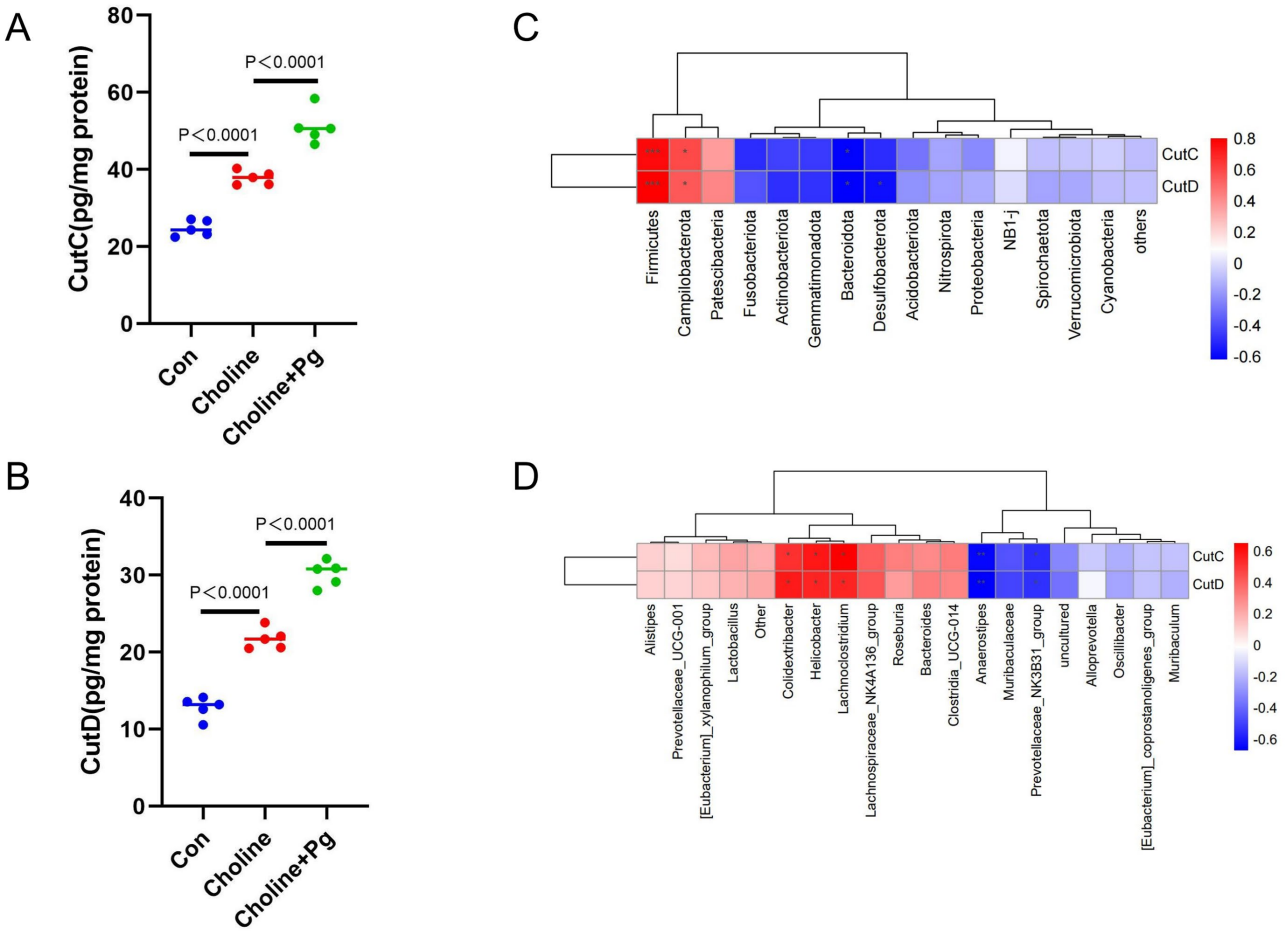
Expression of choline-TMA lyase (*cutC/cutD*) in gut microbiota of Con, Choline, and Choline + *P*g groups. **(A)** Cecal cutC protein concentration; **(B)** Cecal cutD protein concentration; **(C)** Spearman correlation coefficient test between top 20 phyla and cutC/cutD parameters; **(D)** Spearman correlation coefficient test between top 20 genera and cutC/cutD parameters. Data are expressed as mean ± SD (*n* = 5 mice per group). *p < 0.05, **p < 0.01.

As demonstrated in Figures 6A,B, cecal *cutC*/*cutD* proteins were significantly increased in the Choline group compared with the Con and further enhanced in the Choline + *P*g group. Correlation analysis at the phylum level showed positive correlation between *cutC/cutD* levels and *Firmicutes* and *Campilobacterota*, but negative correlation with *Bacteroidota* and *Desulfobacterota* (Figure 6C). At the genus level, *cutC/cutD* expression positively correlated with *Colidextribacter, Helicobacter,* and *Lachnoclostridium*, but negatively correlated with *Anaerostipe*s and *Prevotellaceae_NK3B31_group*, which are consistent with taxa linked to elevated TMA/TMAO metabolism (Figure 6D).

## 4 Discussion

Previous research by our group found that experimental periodontitis aggravated the progression of atherosclerosis in *Apoe^−/−^* mice, which may be related to elevated levels of TMAO in circulation (Xiao et al., 2021). NSPT markedly mitigates plasma TMAO concentrations in *Apoe^−/−^* mice and restores TMAO metabolic homeostasis to a near-physiological state (Huang et al., 2025). However, whether oral *Pg* is an independent factor in the increase in plasma TMAO levels remains unknown. The present study further confirms the direct and independent regulatory role of *Pg* in intestinal microbial TMA-related functional metabolism, which is mediated by the remodeling of the intestinal microbial community structure and enhancement of choline–TMA lyase activity.

Wild-type C57BL/6J mice were used to investigate the effects of *Pg* on TMAO metabolism and the underlying gut microbiota-related mechanisms. It was found that in wild-type C57BL/6J mice without inherent metabolic disorders, oral application of *Pg* significantly increased plasma TMAO levels, accompanied by reduced *α*-diversity of gut microbiota, altered community composition related to TMA metabolism, and impaired intestinal barrier function. Choline is an essential nutrient for the human body, and its metabolism can be divided into four main pathways, which are involved in the synthesis of acetylcholine, betaine, phospholipids, and TMA, respectively. In the gut, microbial production of TMA is initiated from dietary choline, which is catalyzed by the specific glycyl radical enzyme (GRE) choline-TMA lyase (cutC) and its cognate GRE activating enzyme (CutD). The generated TMA is further absorbed into the circulation and metabolized to TMAO in the liver. Based on the results of Experiment 1 and the Choline/TMA/TMAO pathway, we inoculated *Pg* into the oral cavity of mice fed a high-choline diet via bacterial smearing to observe the effects of *Pg* on intestinal homeostasis and the parameters evaluated in this study.

In mice fed a high-choline diet to promote choline/TMA/TMAO metabolism, additional oral administration of *P*g further disrupted TMA metabolism and significantly increased plasma levels of TMAO and TMA. Meanwhile, the composition of the intestinal microbiota was altered in a manner consistent with these changes in TMA metabolism. The significantly upregulated expression of choline–TMA lyase (*cutC/cutD*) in the cecum further proved that orally administered *Pg* may induce abnormal TMA metabolism by regulating the metabolic enzymes of the intestinal flora.

The gut microbiota plays a pivotal role in TMAO synthesis by converting dietary choline and l-carnitine into TMA (Craciun and Balskus, 2012). The 16S rRNA sequencing results from this study revealed that *Pg* infection increased the *Firmicutes* to *Bacteroidota* ratio (F/B ratio), consistent with previous observations (Kato et al., 2018; Kato et al., 2018; Nakajima et al., 2015). Notably, *Firmicutes* harbor choline–TMA lyase (*cutC/cutD*) genes and are efficient in choline catabolism, whereas *Bacteroidota* lack this enzyme and inhibit TMA production (Craciun and Balskus, 2012). Thus, an increased F/B ratio in *Pg*-infected mice likely enhances intestinal TMA synthesis, which is subsequently oxidized by hepatic FMOs into plasma TMAO (Alibrandi et al., 2021).

At the genus level, *Helicobacter*, which was more abundant in the *P*g group, has been linked to metabolic disorders and atherosclerosis (Cuba et al., 2025; Shi et al., 2022). In contrast, the decreased abundance of *Bacteroides*, a key genus within the Bacteroidota phylum, may further promote TMAO accumulation. This is consistent with clinical evidence showing an inverse correlation between *Bacteroides* levels and plasma TMAO concentrations in CVD patients (Yoshida et al., 2020).

In mice fed a high-choline diet, *Pg* administration significantly increased the abundance of *Lachnoclostridium*. This genus harbors a high copy number of the *cutC* gene and has been associated with accelerated atherosclerosis in humans (Cai et al., 2022). Collectively, these results indicate that *Pg* selectively enriches TMA-producing bacteria in the gut, thereby enhancing TMAO production.

*CutC/cutD* is the rate-limiting enzyme that catalyzes the cleavage of choline into TMA, and its activity directly determines intestinal TMA production efficiency (Craciun and Balskus, 2012; Craciun et al., 2014). In the present study, intestinal *cutC/cutD* protein levels were elevated in mice maintained on a high-choline diet, and these levels were significantly upregulated following oral administration of *Pg*. This finding confirms that *Pg* can enhance the expression of the intestinal *Cut* family enzymes. Accumulating evidence suggests that gut microbiota possess heterogeneous capacities for choline utilization. Specifically, bacteria belonging to *Firmicutes* can catalyze choline cleavage to produce TMA, whereas choline-TMA lyase is nearly absent in the phylum *Bacteroidota* (Craciun et al., 2014). Compared with the Choline group, the Choline + *P*g group exhibited a significantly higher abundance of *Bacteroidota* but a lower abundance of *Firmicutes*. Notably, increased expression of choline-TMA lyase was observed in the Choline + *P*g group. This finding seems contradictory to existing evidence, which indicates that TMA lyase is predominantly enriched in *Firmicutes* and minimally expressed in *Bacteroidota*. Cai et al. (2022) identified 13 bacterial genomes carrying the cut gene cluster across 14 intestinal genera, among which *Lachnoclostridium* and *Clostridium* had the highest abundance of *cutC*. As a key rate-limiting enzyme in TMA biosynthesis, the upregulation of *cutC/cutD* is a hallmark of enhanced TMA-producing capacity (Cai et al., 2022). This is consistent with the changes in TMA concentration and microbial community observed in the present study. Collectively, this indicates that *Pg* can skew the gut microbiota toward a “TMA-high metabolic” phenotype.

Targeting this mechanism may facilitate the development of multifaceted combinatorial therapeutic strategies for periodontitis-associated systemic metabolic disorders. First, NSPT regimens should be optimized to attenuate *Pg* colonization, combined with microbiota-targeted interventions (e.g., probiotic supplementation and fecal microbiota transplantation) to reduce the abundance of TMA-producing microorganisms. Next, building on the strategy of Benson et al. of fluoromethylcholine-mediated targeting and inhibition of intestinal microbial *cutC* enzyme (Benson et al., 2023) may be done to develop inhibitors specifically targeting *Pg*-mediated TMA biosynthesis pathways. FMO3 inhibitors should be incorporated to block hepatic TMA-to-TMAO conversion. Notably, Ma et al. demonstrated that oral berberine administration in animal models reduced TMAO levels by inhibiting the activity of gut microbial *cutC* and hepatic FMO enzymes (Ma et al., 2022). Finally, dietary interventions should aim at reducing the intake of TMA biosynthetic precursors (e.g., choline and l-carnitine) for synergistic TMAO reduction (Luqman et al., 2024). Collectively, these strategies may effectively mitigate the risk of periodontitis-related CVDs by modulating the gut–liver axis metabolism, thereby offering a novel therapeutic paradigm for periodontitis-associated systemic metabolic disorders.

This study provides evidence that *Pg* elevates plasma TMAO levels by modulating the gut microbiota composition, enhancing choline–TMA lyase (*cutC/cutD*) activity, and impairing intestinal barrier integrity. These findings reveal a novel oral–gut–metabolic axis and offer new insights into the contribution of periodontal pathogens to systemic metabolic disorders.

## Data Availability

The raw data supporting the conclusions of this article will be made available by the authors, without undue reservation.
