# Supplementary figures and images for: Porphyromonas gingivalis drives trimethylamine-N-oxide accumulation via modulation of gut microbial trimethylamine lyase in mice
Source: Front Microbiol. 2026 Apr 7;17:1786725. doi: 10.3389/fmicb.2026.1786725 (PMC13095576; doi:10.3389/fmicb.2026.1786725)

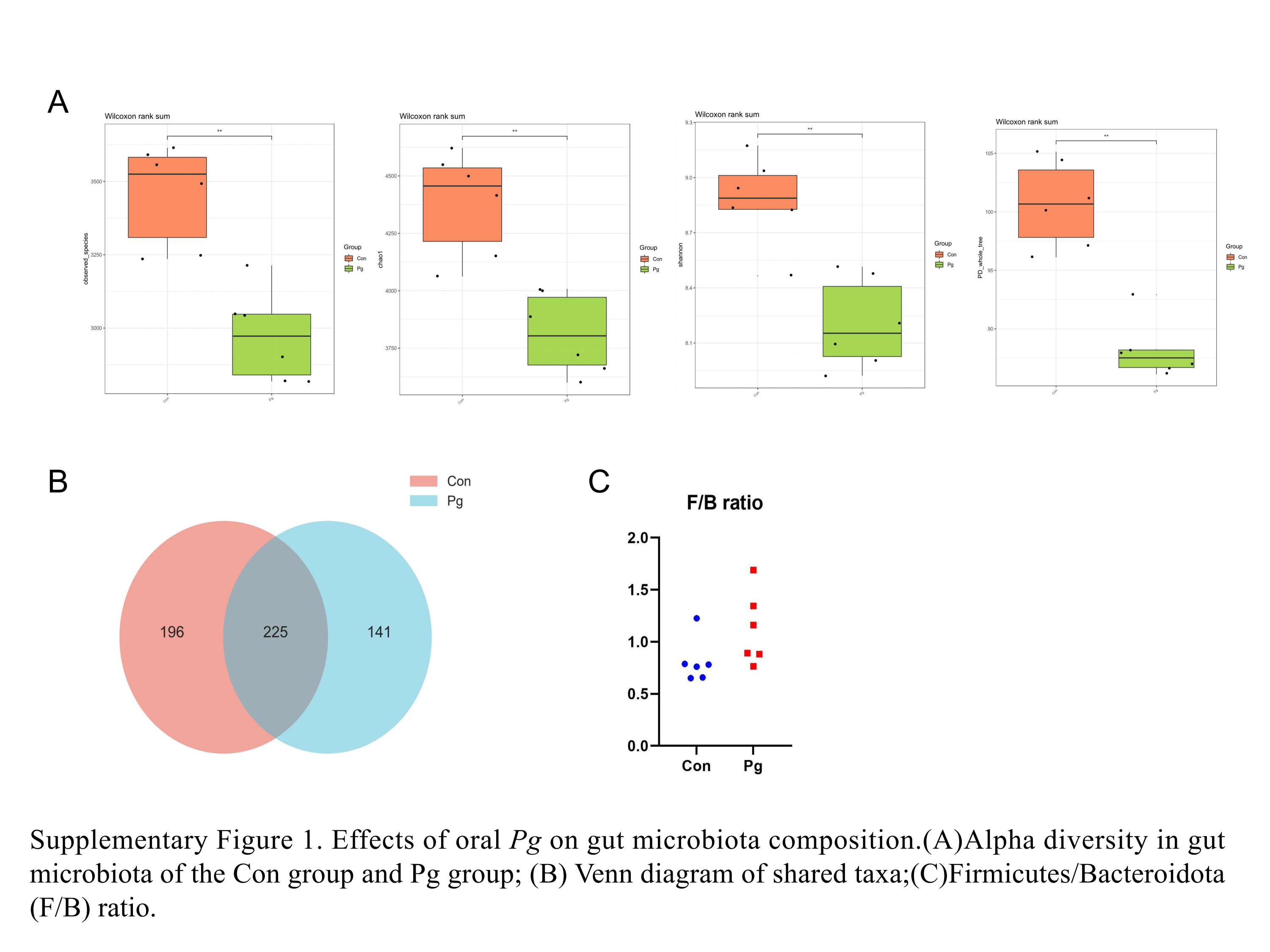

Supplement: Supplementary file 1 [file Image_1.tif]

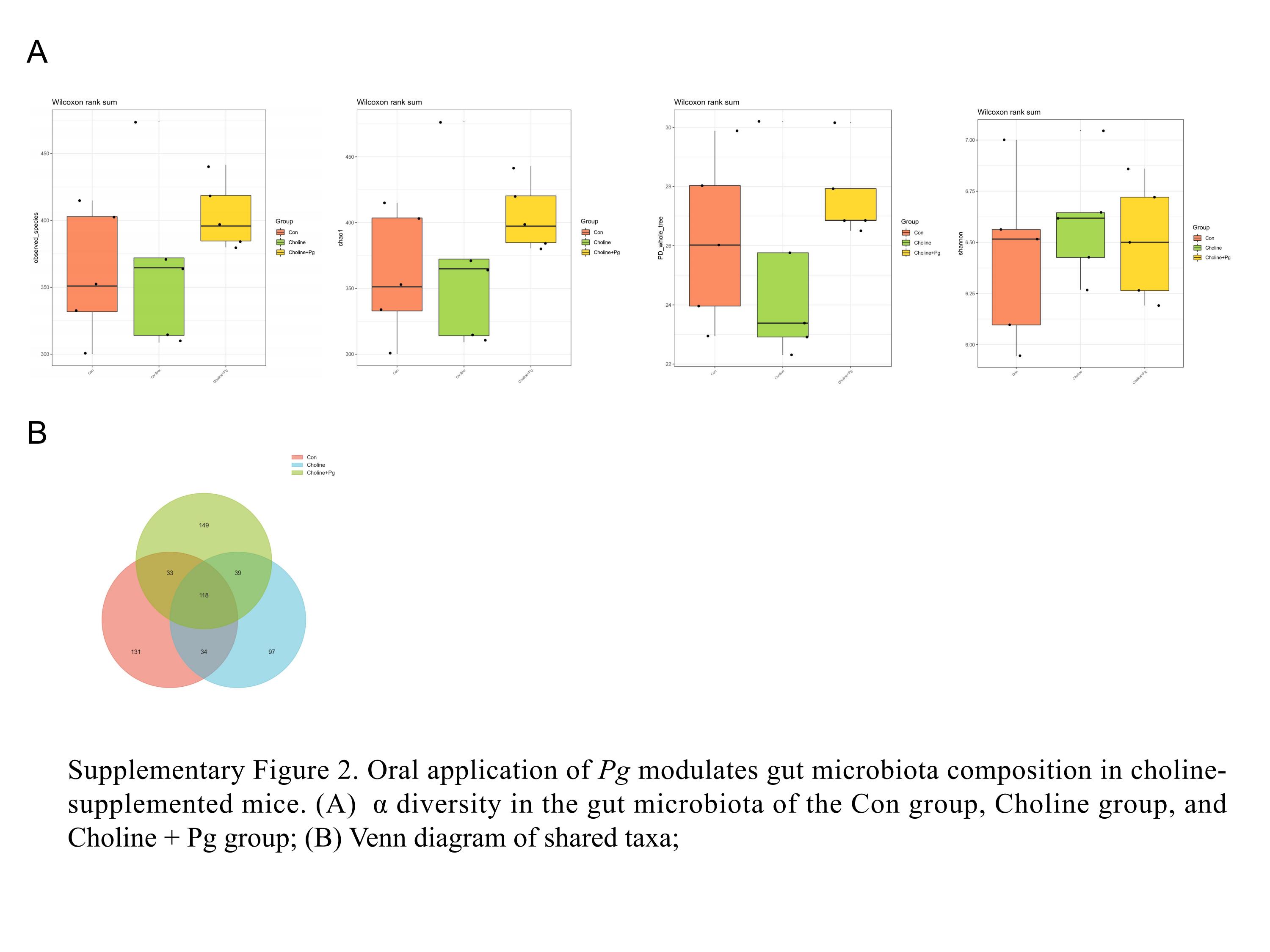

Supplement: Supplementary file 2 [file Image_2.tif]
